# Role of liver stiffness measurements in patients who develop hepatocellular carcinoma after clearance of the hepatitis C virus

**DOI:** 10.1007/s10396-021-01188-x

**Published:** 2022-02-07

**Authors:** Yoshinori Gyotoku, Ryosaku Shirahashi, Toshikuni Suda, Masaya Tamano

**Affiliations:** Department of Gastroenterology, Dokkyo Medical University Saitama Medical Center, 2-1-50 Minami-Koshigaya, Koshigaya-shi, Saitama, 343-8555 Japan

**Keywords:** Hepatitis C, hepatocellular carcinoma, Direct-acting antiviral therapy, Shear wave elastography

## Abstract

**Purpose:**

To measure changes in liver stiffness over time due to direct-acting antiviral (DAA) therapy in hepatitis C patients using shear wave elastography (SWE).

**Methods:**

Patients with hepatitis C treated with DAA therapy in a university medical center between July 2015 and April 2020 were evaluated. Shear wave velocity (Vs) of the liver was measured using SWE. Alanine aminotransferase (ALT), platelet count, and α-fetoprotein (AFP) were measured at the same time, and the FIB-4 index was estimated. Absence of hepatocellular carcinoma was confirmed at baseline and end of therapy. Imaging was then performed every 6 months. Patient characteristics were compared between patients who did and did not develop carcinoma.

**Results:**

The mean age of the 229 patients (93 men) was 65.6 years. Eight patients developed carcinoma during follow-up (mean 32.6 ± 19.5 months). Significant differences were found between the groups in terms of AFP, platelet count, and Fib-4 index at baseline; the pre-treatment data had the best relationship with hepatocarcinogenesis. Mean Vs decreased significantly during DAA therapy, and then decreased further. Liver stiffness 6 months after treatment ended had the best relationship with hepatocarcinogenesis.

**Conclusion:**

In patients with a sustained virological response, risk of developing cancer can be predicted by measuring Vs approximately 6 months after treatment.

## Introduction

Significant advances have been made in the treatment of hepatitis C with the advent of direct-acting antiviral agents (DAAs). DAA therapy has milder side effects than IFN therapy, and there is a high rate of sustained virological response (SVR) [1–5]. When SVR is achieved with DAA therapy, cancer inhibition similar to that with IFN therapy is seen [6–8]. High ALT and AFP values and low platelet counts are reported to be risk factors for cancer after SVR [9], but they are analyzed based on pre- and post-treatment clinical data.

At our hospital, we see many patients in whom SVR was achieved with DAA therapy at other institutions. In such cases, since we do not know the blood test findings at the start and end of DAA therapy, it is a struggle to infer the cancer risk of the patient.

Shear wave elastography (SWE) is a technique that is used to assess stiffness of the liver by measuring the propagation velocity of shear waves (Vs) generated in liver tissue. Vs measurements with SWE are effective in diagnosing liver fibrosis in patients with hepatitis C [10].

A previous study showed that, in patients with hepatitis C, measurements of liver stiffness by SWE were higher in the naïve group compared to the SVR group, probably due to hepatitis activity [11]. In addition, compared with pre-treatment levels, an improvement in liver stiffness was reported in patients with hepatitis C who achieved SVR by treatment with DAAs [12]. Further, this improvement in liver stiffness was observed to be more noticeable in patients with hepatitis C with advanced fibrosis [13, 14].

In this study, the focus was on liver stiffness measured by SWE that changes over time due to DAA therapy in patients with hepatitis C. Hepatitis C patients in whom SVR is achieved will continue to increase in the future, and if liver stiffness after the end of DAA therapy were shown to affect carcinogenesis, it would contribute greatly to screening these patients for hepatocellular carcinoma (HCC).

## Patients and methods

### Patients

The subjects included 355 patients at a university medical center who were diagnosed with hepatitis C and treated with DAAs between July 2015 and April 2020. Patients with autoimmune disease, chronic heart disease, collagen disease, decompensated liver cirrhosis, or HCC were excluded from the study. Patients with a history of HCC were also not included in this study. Similarly, patients diagnosed with fatty liver on ultrasonography or those with chronic alcohol consumption history (alcohol intake ≥ 20 g/day) were also excluded.

### Shear wave velocity measurements

A LOGIQ E9 multi-purpose ultrasound system (GE Healthcare, Milwaukee, WI, USA) was used to measure Vs (m/s) by SWE. Visualization of segment 5 of the liver was performed through an intercostal space. Patients abducted the right arm in the supine position on a bed. Measurements of Vs were performed while patients held their breath. The region of interest (ROI) was fixed 1–2 cm under the surface of the liver. Adjustments were made to the system so that sample volume depth was less than or equal to 4 cm. Vs measurements were calculated automatically by the system, and results were considered reliable when the measurement success rate was > 80% from ten successful shots.

For all patients, the duration of DAA treatment was 8–12 weeks. SWE was performed before initiating treatment (baseline), at the end of treatment (EOT), 12 weeks after EOT (follow-up 12), and 24 weeks after EOT (follow-up 24).

### Clinical parameters

The clinical parameters that were obtained on the same day that SWE was performed were compared. In addition to Vs, the clinical parameters measured included α-fetoprotein (AFP), alanine aminotransferase (ALT), and platelet count. Parameter measurements were conducted on the same days as SWE. The fibrosis-4 (FIB-4) index for liver fibrosis was estimated using patient age and the values obtained for ALT, serum aspartate transaminase (AST), and platelet count.

### Comparison of non-carcinogenic and carcinogenic groups

In all of the patients, it was confirmed on abdominal ultrasound, CT scan, or Gd-EOB-DTPA-enhanced MRI (EOB-MRI) that there were no HCC complications at baseline and EOT. Abdominal ultrasound was performed every 6 months after the end of therapy, and if a liver tumor was seen, contrast CT or EOB-MRI was performed.

Patient characteristics at the start of treatment were compared between the non-carcinogenic group, in which HCC was not seen during the observation period, and the carcinogenic group, in which HCC was seen during the observation period. In addition, factors for which significant differences were seen in data at the start of treatment were confirmed at EOT, follow-up 12, and follow-up 24.

### Statistical analysis

The data for clinical parameters and liver stiffness are expressed as means ± standard deviation (SD). A paired Wilcoxon test was used to compare each parameter before and after starting treatment. The diagnostic performances of liver stiffness and other clinical parameters for predicting the presence of HCC were evaluated using receiver operating characteristic (ROC) curve analyses. The area under the ROC curve (AUROC) was used to assess diagnostic accuracy.

## Results

### Patient characteristics at the start of treatment

**Fig. 1:**
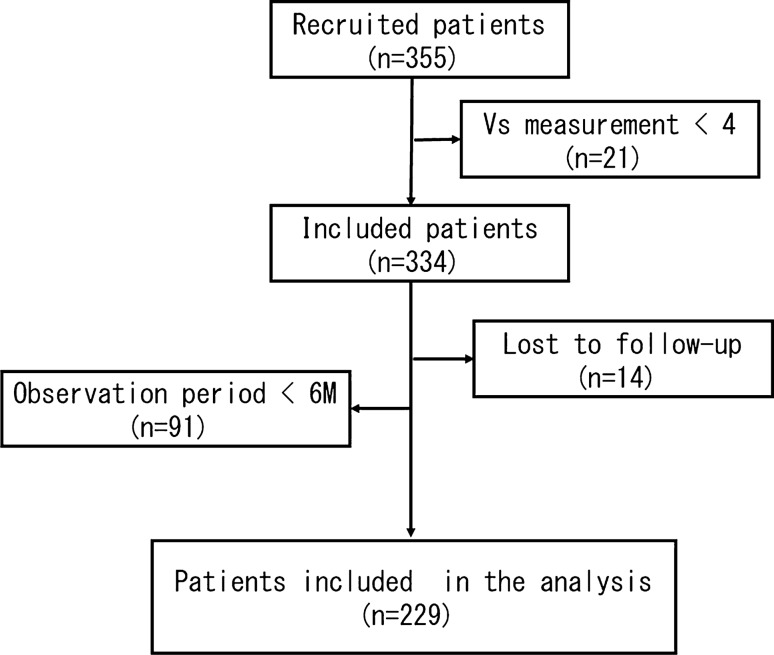
Flowchart of the study. A total of 355 consecutive hepatitis C patients were recruited. After excluding 21 patients with less than four Vs measurements, 12 patients lost to follow-up, and 91 patients with observation periods of less than 6 months, 229 patients were included in the study

A total of 355 patients with hepatitis C were recruited for the study. After excluding 21 patients for whom Vs was not measured all three times, 14 patients lost to follow-up, and 91 patients for whom the observation period was less than 6 months, the investigation included 229 cases (Fig. 1).

Table 1 shows patient characteristics at the start of treatment. The mean age was 65.6 years, 93 were men, and 136 were women. The DAAs selected for treating these 229 patients were sofosbuvir/ribavirin in 71 patients, SOF/ledipasvir (LDV) in 58 patients, ombitasvir/paritaprevir/ritonavir in 41 patients, elbasvir/pibrentasvir in 30 patients, glecaprevir/pibrentasvir in 24 patients, ombitasvir/paritaprevir/ritonavir/ribavirin in three patients, and daclatasvir/asunaprevir in two patients. A total of 42 patients had previously received interferon (IFN) therapy. The DAA treatment period was 8–12 weeks, depending on the type of drug.

**Table 1:** Patient characteristics (*n* = 229)

Age (y)	65.6 ± 12.6 (21–84)
Sex (male/female)	93/136
Genotype (1b/2a/2b/1a + 2b/3a + 3b/Unknown)	121/54/25/1/2/26
Interferon (yes/no/unknown)	42/153/34
HCV RNA (LogIU/ml)	5.81 ± 0.87 (2.5–7.3)
ALT (IU/L)	55.5 ± 46.8 (6–426)
GGT (U/L)	49.5 ± 55.3 (9–575)
Total bilirubin (mg/dl)	0.85 ± 0.63 (0.1–9)
Alb (g/dl)	4.16 ± 0.37 (2.9–5.3)
WBC (10^4^/mm^3^)	5.11 ± 1.69 (2–12.2)
Hb (g/dl)	13.8 ± 1.50 (10–18.1)
Plt (10^4^/mm^3^)	16.7 ± 6.19 (4.1–36.1)
PT (%)	98.5 ± 16.6 (27–147)
AFP (ng/ml)	9.49 ± 19.1 (0.6–140.5)
Vs (m/s)	1.58 ± 0.26 (1.03–2.56)
Fib-4 index	3.49 ± 2.76 (0.30–19.1)
Observation period (M)	32.6 ± 19.5(6–74)
Hepatocellular carcinoma (Yes/No)	8/211

*HCV* hepatitis C virus, *ALT* alanine aminotransferase, *GGT* γ-glutamyltransferase, *WBC* white blood cell count, *Plt* platelet count, *Alb* serum albumin, *PT%* prothrombin time, *AFP* alpha-fetoprotein, *Vs* shear wave velocity, *Fib-4 index* fibrosis-4 index

Mean ALT was 55.5 IU/L, showing a moderate increase. In contrast, total bilirubin, albumin, platelet count, and prothrombin activity were within the standard range. Mean AFP was 9.49 ng/ml, mean Vs was 1.58 m/s, and mean Fib-4 index was 3.49.

The mean observation period for the 229 patients was 32.6 ± 19.5 (6–74) months, and hepatocellular carcinoma was seen in eight of the 229 patients.

### Patient characteristics in non-carcinogenic group and carcinogenic group

Table 2 shows the characteristics of the 229 patients in the non-carcinogenic group and the eight patients in the carcinogenic group. There was a trend toward higher age in the carcinogenic group compared with the non-carcinogenic group, but the difference was not significant (*p* = 0.1742). Similarly, there was a trend toward more men in the carcinogenic group, but no significant difference was seen (*p* = 0.5820).

**Table 2:** Patient characteristics in the non-carcinogenic group and carcinogenic group at baseline

	Non-carcinogenic group (*n* = 211)	Carcinogenic group (*n* = 8)	*p* value
Age (y)	65.3 ± 12.7 (21–84)	72.1 ± 6.8 (64–84)	0.1742
Sex (male/female)	89/132	4/4	0.5820
ALT (IU/L)	54.2 ± 45.9 (6–426)	99.3 ± 56.4 (37–187)	0.0095
GGT (U/L)	49.2 ± 56.0 (9–575)	57.3 ± 28.3 (33–111)	0.1293
Total bilirubin (mg/dl)	0.84 ± 0.63 (0.1–9)	1.01 ± 0.50 (0.7–2.1)	0.1980
Alb (g/dl)	4.2 ± 0.4 (2.9–5.3)	3.9 ± 0.3 (3.5–4.4)	0.0744
WBC (10^4^/mm^3^)	5.12 ± 1.70 (2–12.2)	4.59 ± 1.26 (2.5–5.7)	0.7181
Hb (g/dl)	13.7 ± 1.50 (10–18.1)	14.0 ± 1.13 (12.2–15.7)	0.6602
Plt (10^4^/mm^3^)	17.0 ± 6.2 (4.1–36.1)	11.3 ± 2.6 (8.6–15.2)	0.0059
PT% (%)	98.8 ± 16.7 (27–147)	90.7 ± 11.8 (78–106)	0.1444
AFP (ng/ml)	8.0 ± 14.4 (0.6–140.5)	51.5 ± 52.5 (8.9–139.9)	0.0003
Vs (m/s)	1.58 ± 0.26 (1.03–2.56)	1.86 ± 0.20 (1.52–2.26)	0.0036
Fib-4 index	3.38 ± 2.71 (0.30–19.11)	6.87 ± 2.40 (3.43–10.13)	0.0006
Observation period (M)	33.0 ± 19.4 (6–74)	21.3 ± 17.6 (6–58)	0.1193

*ALT* alanine aminotransferase, *GGT* γ-glutamyltransferase, *WBC* white blood cell count, *Plt* platelet count, *Alb* serum albumin, *PT%* prothrombin time, *AFP* alpha-fetoprotein, *Vs* shear wave velocity, *Fib-4 index* fibrosis-4 index

Compared with the non-carcinogenic group, ALT, AFP, Vs, and Fib-4 index were significantly higher in the carcinogenic group (*p* = 0.0095, *p* = 0.0003, *p* = 0.0036, and *p* = 0.0006, respectively). The platelet count was significantly lower in the carcinogenic group than in the non-carcinogenic group (*p* = 0.0059). Albumin and prothrombin activity tended to be lower in the carcinogenic group, but the differences were not significant (*p* = 0.0744 and *p* = 0.1444, respectively). The mean time from the end of treatment to the development of cancer was 21.3 months.

### Clinical parameters in the non-carcinogenic group and carcinogenic group

ALT, platelet count, AFP, VS, and Fib-4 index are shown in Table 3 for the carcinogenic and non-carcinogenic groups. At EOT, significant differences were seen in all five parameters. Similar to the results at EOT, significant differences were seen in four parameters all but ALT at follow-up 12 and 24.

**Table 3:** Clinical parameters in the non-carcinogenic group and carcinogenic group

	Non-carcinogenic group (*n* = 211)	Carcinogenic group (*n* = 8)	*p* value
ALT (U/L)			
EOT	54.2 ± 45.9 (6–426)	99.3 ± 56.4 (37–187)	0.0095
Follow-up 12	19.8 ± 14.4 (7–95)	32.8 ± 37.4 (12–122)	0.2271
Follow-up 24	17.2 ± 11.5 (4–123)	24.7 ± 20.4 (8–64)	0.2495
Plt (10^4^ /mm^3^)			
EOT	17.0 ± 6.2 (4.1–36.1)	11.3 ± 2.6 (8.6–15.2)	0.0059
Follow-up 12	18.5 ± 7.0 (3.6–47.2)	13.3 ± 4.6 (7.9–20.7)	0.0261
Follow-up 24	18.2 ± 6.5 (2.9–38.6)	13.6 ± 3.6 (9.7–19.6)	0.0469
AFP (ng/ml)			
EOT	8.0 ± 14.4 (0.6–140.5)	51.0 ± 60.6 (8.9–139.9)	0.0003
Follow-up 12	4.1 ± 2.8 (0.6–14.8)	9.0 ± 4.5 (5–17.4)	0.0007
Follow-up 24	3.8 ± 2.5 (0.6–18.7)	8.8 ± 5.3 (3.7–16.5)	0.0019
Vs (m/s)			
EOT	1.58 ± 0.26 (1.03–2.56)	1.75 ± 0.22 (1.35–2.15)	0.0036
Follow-up 12	1.49 ± 0.25 (1.01–2.25)	1.66 ± 0.15 (1.49–1.87)	0.0184
Follow-up 24	1.44 ± 0.24 (0.95–2.25)	1.69 ± 0.121( 1.50–1.95)	0.0308
Fib-4 index			
EOT	3.38 ± 2.71 (0.30–19.11)	6.87 ± 2.40 (3.43–10.13)	0.0006
Follow-up 12	2.55 ± 1.914( 0.28–12.68)	4.49 ± 2.72 (1.64–10.44	0.0067
Follow-up 24	2.53 ± 1.75 (0.01–15.52)	4.41 ± 1.84( 2.44–7.55)	0.0048

*ALT* alanine aminotransferase, *Plt* platelet count, *AFP* alpha-fetoprotein, *Vs* shear wave velocity, *Fib-4 index* fibrosis-4 index

### Changes in Vs from baseline to follow-up 24

**Fig. 2:**
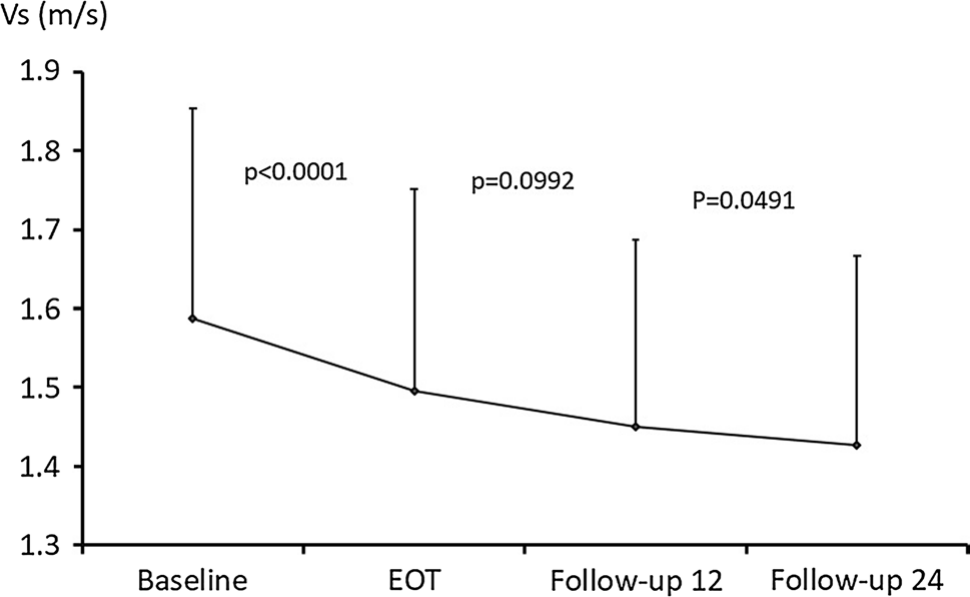
Changes over time in Vs. Vs decreases over time. It is 1.59 ± 0.27 m/s at baseline, 1.50 ± 0.26 m/s at EOT, 1.45 ± 0.24 m/s at follow-up 12, and 1.43 ± 0.02 m/s at follow-up 24, but it does not reach the value for a healthy liver (1.2 m/s). *Baseline* before starting treatment, *EOT* end of treatment, *follow-up 12* 12 weeks after EOT, *follow-up 24* 4 weeks after EOT

Figure 2 shows the changes in Vs from baseline to follow-up 24. Vs measurements decreased over time, measuring 1.59 ± 0.27 m/s at baseline, 1.50 ± 0.26 m/s at EOT, 1.45 ± 0.24 m/s at follow-up 12, and 1.43 ± 0.02 m/s at follow-up 24 (*p* < 0.0001, *p* = 0.0992, and *p* = 0.0491, respectively).

### Eight carcinogenic patients

We show the list of eight carcinogenic patients in Table 4. Only one patient (No. 7) had two tumors, while the other seven patients had a single tumor. All eight patients were treated and had a good clinical course.

**Table 4:** List of eight carcinogenic patients

No	Sex	Age (years)	AFP (ng/ml)	Vs (m/s)			Tumor size (mm)
				EOT	Follow-up 12	Follow-up 24	
1	Male	74	13.7	1.81	1.86	1.95	30
2	Female	78	137.5	2.15	1.87	1.65	20
3	Female	71	9.5	1.71	1.55	1.50	10
4	Male	64	12.3	1.55	1.49	1.53	12
5	Female	84	8.9	1.71	1.63	1.87	20
6	Female	65	35.3	1.87	1.67	1.92	50
7	Male	67	54.5	1.35	1.45	1.35	10
8	Male	74	139.9	1.85	1.79	1.76	15

*AFP* alpha-fetoprotein, *Vs* shear wave velocity

### Receiver operating characteristic curve analyses

**Fig. 3:**
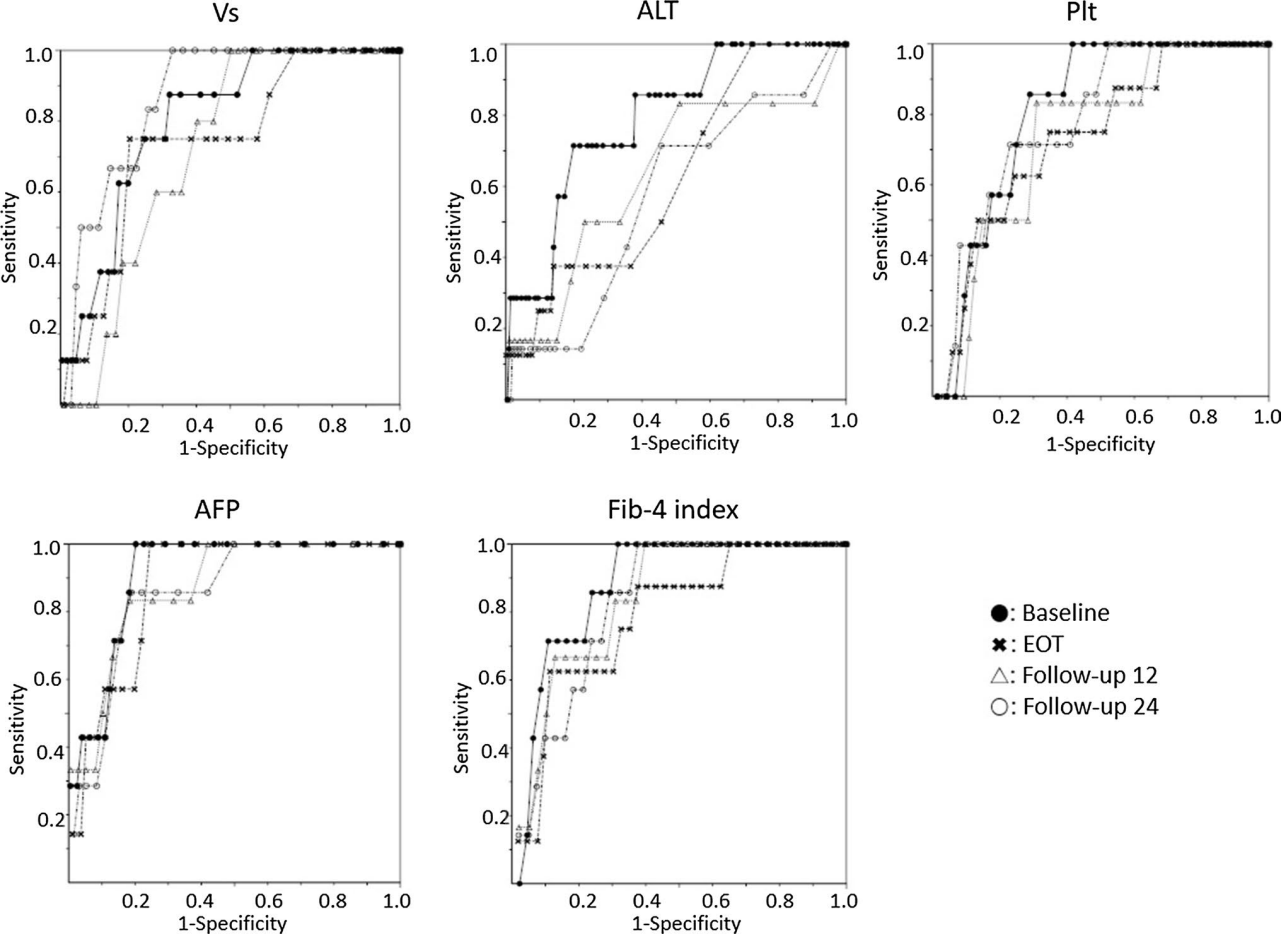
Receiver operating characteristic curves for Vs, ALT, AFP, Fib-4 index, and platelet count at each time point. The area under the receiver operating characteristic curve (AUROC) of Vs is 0.80317 at baseline, 0.74548 at EOT, 0.72147 at follow-up 12, and 0.86041 at follow-up 24; the largest at follow-up 24. Similarly, that of ALT is 0.78798 at baseline, 0.62557 at EOT, 0.63789 at follow-up 12, and 0.56628 at follow-up 24; that of AFT is 0.90746 at baseline, 0.87760 at EOT, 0.87261 at follow-up 12, and 0.84929 at follow-up 24; that of Fib-4 index is 0.87395 at baseline, 0.78252 at EOT, 0.83849 at follow-up 12, and 0.82956 at follow-up 24; and that of platelet count is 0.80608 at baseline, 0.73162 at EOT, 0.73840 at follow-up 12, and 0.78640 at follow-up 24. The AUROC is the largest at baseline for all parameters. *Baseline* before starting treatment, *EOT* end of treatment, *follow-up 12* 12 weeks after EOT, *follow-up 24* 4 weeks after EOT

The ROC curves for Vs, ALT, AFP, Fib-4 index, and platelet count at each time point are shown in Fig. 3. The AUROC of Vs was 0.80 at baseline, 0.75 at EOT, 0.72 at follow-up 12, and 0.86 at follow-up 24. Thus, it was the largest at follow-up 24. Similarly, that of ALT was 0.79 at baseline, 0.63 at EOT, 0.64 at follow-up 12, and 0.57 at follow-up 24; that of AFP was 0.91 at baseline, 0.88 at EOT, 0.87 at follow-up 12, and 0.85 at follow-up 24; that of Fib-4 index was 0.87 at baseline, 0.78 at EOT, 0.84 at follow-up 12, and 0.83 at follow-up 24; and that of platelet count was 0.81 at baseline, 0.73 at EOT, 0.74 at follow-up 12, and 0.79 at follow-up 24. The AUROC was the largest at baseline for all parameters.

## Discussion

Hepatitis C patients are reported to have sustained changes in protein expression due to epigenetic changes in hepatocytes even after SVR, and these changes contribute to the development of liver cancer [15]. Therefore, identification of carcinogenic risk factors after SVR with DAA therapy and the creation of surveillance systems are issues that need to be addressed.

Advanced age, progression of hepatic fibrosis, male sex, and AFP level have been shown to be independent risk factors for hepatocellular carcinogenesis in hepatitis C patients [16–19]. In this study, although there was no significant difference in age or sex at baseline between the carcinogenic and non-carcinogenic groups, this was likely due to the small number of people in the carcinogenic group. Significant differences were shown between the groups in terms of AFP level, platelet count, and Fib-4 index, which are indicators of hepatic fibrosis, at baseline.

A previous study using SWE reported that Vs was approximately 1.2 m/s in healthy livers.^11^ Although the mean Vs at baseline in this study was 1.56 ± 0.26 m/s, Vs decreased significantly to 1.50 ± 0.26 m/s over the 8–12 weeks of DAA therapy. Vs at follow-up 12 and 24 showed significant decreases to 1.45 ± 0.24 m/s and 1.43 ± 0.24 m/s, respectively.

Vs decreased over time from baseline to follow-up 24, but it did not reach the level found in healthy livers. When SVR is achieved in hepatitis C patients, it is reported to take 3 years for histological improvement in fibrosis [20]. Liver stiffness, inferred using Vs, is influenced not only by the degree of liver fibrosis but also by the degree of necroinflammatory activity [21, 22]. Both inflammation and fibrosis of the liver contribute to baseline Vs, and Vs is thought to more accurately show fibrosis of the liver with the passage of time at EOT, follow-up 12, and follow-up 24.

As shown in Table 2, a comparison of patient characteristics at baseline showed higher ALT, AFP, Vs, and Fib-4 index, and a lower platelet count, in the carcinogenic group than in the non-carcinogenic group. This shows that liver inflammation, regeneration, and fibrosis at baseline contribute to carcinogenesis. As shown in Table 3, comparisons of these five parameters at each time point showed significant differences in all but ALT.

Investigation of the AUROC of ALT, AFP, Vs, Fib-4 index, and platelet count at each time point revealed that the AUROC was the largest at baseline for ALT, AFP, Fib-4 index, and platelet count. In contrast, the AUROC was the largest at follow-up 24 for Vs.

These results show that, for ALT, AFP, Fib-4 index, and platelet count, pre-treatment data have the best relationship with hepatocarcinogenesis, whereas liver stiffness measured using SWE has the best relationship with hepatocarcinogenesis at 6 months after EOT.

Vs at follow-up 24 and later is very interesting, but, unfortunately, the number of patients in whom Vs was measured 6 months and beyond after EOT was very small in this study, and so this could not be investigated.

## Limitations

This retrospective study was conducted at a single institution. Although 355 patients were enrolled in this study, data from only 229 patients met the criteria for inclusion in the study. Cancer developed in only eight patients, and so independent factors contributing to carcinogenesis could not be tested.

## Conclusions

This study demonstrated that, when an SVR patient whose pre- and post-treatment data are not known presents for initial examination, the patient’s risk of developing cancer can be predicted by measuring Vs approximately 6 months after treatment. The number of hepatitis C patients achieving SVR will continue to increase in the coming years, and this result will contribute greatly to HCC screening in these patients.
